# Targeting FOXP3 Tumor-Intrinsic Effects Using Adenoviral Vectors in Experimental Breast Cancer

**DOI:** 10.3390/v15091813

**Published:** 2023-08-25

**Authors:** Alejandro J. Nicola Candia, Matías Garcia Fallit, Jorge A. Peña Agudelo, Melanie Pérez Küper, Nazareno Gonzalez, Mariela A. Moreno Ayala, Emilio De Simone, Carla Giampaoli, Noelia Casares, Adriana Seilicovich, Juan José Lasarte, Flavia A. Zanetti, Marianela Candolfi

**Affiliations:** 1 Instituto de Investigaciones Biomédicas (INBIOMED, UBA-CONICET), Facultad de Medicina, Universidad de Buenos Aires, Buenos Aires C1121A6B, Argentina; alenicola90@gmail.com (A.J.N.C.); adriana.seilicovich@gmail.com (A.S.); 2 Departamento de Química Biológica, Facultad de Ciencias Exactas y Naturales, Universidad de Buenos Aires, Buenos Aires C1121A6B, Argentina; 3 Cátedra de Fisiología Animal, Facultad de Ciencias Veterinarias, Universidad de Buenos Aires, Buenos Aires C1428BFA, Argentina; 4 Program Immunology and Immunotherapy, Centro de Investigación Médica Aplicada (CIMA, CUN), 31008 Pamplona, Spain; ncasares@unav.es (N.C.);; 5 Instituto de Investigación Sanitaria de Navarra (IDISNA), 31008 Pamplona, Spain; 6 Instituto de Ciencia y Tecnología “Dr. Cesar Milstein”, Consejo Nacional de Investigaciones Científicas y Técnicas (CONICET), Saladillo C1440FFX, Buenos Aires, Argentina; flaviazanetti66@hotmail.com

**Keywords:** Foxp3, cell penetrating peptide, gene therapy, breast cancer, chemosensitivity

## Abstract

The regulatory T cell master transcription factor, Forkhead box P3 (Foxp3), has been detected in cancer cells; however, its role in breast tumor pathogenesis remains controversial. Here we assessed Foxp3 tumor intrinsic effects in experimental breast cancer using a Foxp3 binder peptide (P60) that impairs Foxp3 nuclear translocation. Cisplatin upregulated Foxp3 expression in HER2+ and triple-negative breast cancer (TNBC) cells. Foxp3 inhibition with P60 enhanced chemosensitivity and reduced cell survival and migration in human and murine breast tumor cells. We also developed an adenoviral vector encoding P60 (Ad.P60) that efficiently transduced breast tumor cells, reduced cell viability and migration, and improved the cytotoxic response to cisplatin. Conditioned medium from transduced breast tumor cells contained lower levels of IL-10 and improved the activation of splenic lymphocytes. Intratumoral administration of Ad.P60 in breast-tumor-bearing mice significantly reduced tumor infiltration of Tregs, delayed tumor growth, and inhibited the development of spontaneous lung metastases. Our results suggest that Foxp3 exerts protumoral intrinsic effects in breast cancer cells and that gene-therapy-mediated blockade of Foxp3 could constitute a therapeutic strategy to improve the response of these tumors to standard treatment.

## 1. Introduction

The transcription factor Foxp3 is known for its role as a master regulator of regulatory T cell (Treg) function, a dynamic subgroup of CD4+ T lymphocytes that modulate the response of the immune system under physiological and pathological conditions, such as autoimmunity and cancer [1,2]. Foxp3 is composed of three major domains: the proline-rich N-terminal domain (1–97 aa), responsible for transcriptional activation and repression; a central domain (98–260 aa) consisting of a zinc finger and leucine zipper (LZ), involved in the association with other transcription factors; and the forkhead domain (FKH) C-terminal (337–423 aa), responsible for DNA binding. The FKH domain forms homodimers, which contributes to the nuclear translocation of Foxp3, where it binds to target genes [3]. Foxp3 can also form heterodimers with other members of the Foxp family, such as Foxp1, which preserves memory potential and the regulation of Treg development and T-cell exhaustion [4]. Foxp3 is crucial for Treg lineage survival and stability, enabling Treg functionality even in a nutrient-poor and hypoxic environment [5]. In the tumor microenvironment (TME), Foxp3 activation in Tregs generates an exhausting microenvironment, inducing TGF-β and IL-10 expression or downregulating IL-2 [3]. Several strategies have sought to deplete Tregs or block their immunosuppressive function to promote antitumor immunity, without achieving significant therapeutic benefits [6]. Although Tregs were initially identified by their expression of CD25, the expression of this marker is not unique to these cells and it is now known to be a general marker of T cell activation [7]. Studies in which CD25^+^ was targeted by monoclonal antibodies such as basiliximab [8,9], or the use of denileukin diftitox, which directs cytocidal activity of the diphtheria toxin to CD25-expressing cells to facilitate killing [10], did not achieve optimal results; this has been attributed in part to off-target effects by simultaneously reducing effector T cells [11]. Considering that Foxp3 expression is restricted to Tregs, this is an interesting molecule for specifically targeting these cells without affecting other immune cell populations. However, the nuclear localization of Foxp3 impairs the use of blocking antibodies. Cell-penetrating peptides (CPPs) are small peptides (20 aa) capable of crossing the cell membrane [12]. Their capacity to enter cells and excellent biodistribution has generated great interest for the development of novel therapeutic strategies. In this way, P60, a CPP capable of binding Foxp3, has been developed to inhibit the immunosuppressive function of Tregs [13]. P60 showed high affinity for the LZ–FKH domains of Foxp3, preventing Foxp3 nuclear translocation [14]. P60-mediated inhibition of Treg regulatory function stimulates effector T cells, both in vitro and in vivo [15]. In experimental models of kidney and pancreatic cancer, P60 potentiates the efficacy of cytokine-induced NK cell immunotherapy [16].

Although Foxp3 expression within the immune system is virtually restricted to Tregs, this transcription factor has also been detected in several types of normal [17,18] and tumoral epithelial cells, including breast cancer (BRCA) cells [19,20,21,22]. FOXP3 mRNA is significantly upregulated in BRCA biopsies compared with adjacent non-neoplastic breast tissue [23,24]. Although the role of Foxp3 in regulating immunosuppression is very well known, its actions in tumor cells remain uncertain [5]. Although there is consensus regarding the tumor-suppressing role of Foxp3 in the normal mammary gland [25], its role in the pathogenesis of BRCA remains controversial [26,27]. It has been proposed that Foxp3 acts as tumor suppressor in breast tumor cells, inhibiting the expression of protumoral molecules, including HER2 and VEGF, through binding and repression of their promoters [26,27]. In experimental models of BRCA, Foxp3-mediated inhibition of MTA1, CXCR4, and CD44 has been implicated in the inhibition of lung metastases [28]. However, it has also been proposed that tumor cells expressing Foxp3 can activate PD-L1 expression and inhibit CD8+ T cell activity, maintaining an immunosuppressive TME [29]. We have previously reported that systemic administration of P60 in mice bearing HER2+ or TNBC tumors inhibits the expansion of Tregs and improves the efficacy of therapeutic dendritic cell (DC) vaccines [22]. In addition, we have shown that P60 exerts direct antitumor effects, inhibiting tumor growth not only in immune competent mice but also in animals lacking a functional immune system [22], suggesting that Foxp3 exerts intrinsic effects in BRCA cells. In fact, our previous results showed that P60 exerts direct inhibitory effects on the viability, proliferation, and secretion of the immunosuppressive interleukin (IL)-10 in Foxp3^+^ BRCA cells in vitro [22], strengthening the idea that Foxp3 exerts protumoral intrinsic effects in these cells. Here, we intended to further evaluate the effects of Foxp3 on the characteristics that facilitate tumor progression, such as chemoresistance and migration, in human and murine experimental BRCA. 

It is important to consider that systemic inhibition of Foxp3 could induce autoimmunity and other immune-mediated side effects [3,30]. In addition, the low systemic half-life of peptides makes it necessary repeated and frequent administration of them to exert therapeutic effects [22]. P60 expression using gene therapy vectors could address these limitations and increase the local bioavailability of the peptide to be used in vivo. In this sense, adenoviruses have been shown to constitute excellent tools for gene therapy, eliciting high transduction efficiency, ubiquitous tropism, and scalable production systems [31]. Thus, we generated an adenoviral vector encoding P60 (Ad.P60) and evaluated its effects in vitro and in vivo in preclinical BRCA models. 

## 2. Materials and Methods

### 2.1. Drugs

Dulbecco’s Modified Eagle’s Medium (DMEM; 12800017), penicillin–streptomycin, and trypsin–EDTA (0.05%) were obtained from Gibco (Invitrogen, Carlsbad, CA, USA), whereas fetal bovine serum (FBS) was from Natocor (Córdoba, Argentina) and cisplatin was from Microsules (Buenos Aires, Argentina). OCT medium for frozen sections was obtained from Biopack (Buenos Aires, Argentina). Ketamine was obtained from Holliday (Argentina Poniente, Mexico). Xylazine (Kensol) was obtained from König (Buenos Aires, Argentina). 

### 2.2. Cell Lines

BRCA cell lines were grown in Petri dishes containing DMEM with high glucose and sodium bicarbonate, supplemented with 10% FBS and 1% penicillin–streptomycin. Cells were harvested using trypsin–EDTA (0.05%) in PBS and counted with trypan blue. Mouse HER2+ breast carcinoma LM3 cells (*BALB/c*) were generated and kindly provided by Dr. Elisa Bal de Kier Joffé (Hospital Roffo, Buenos Aires Argentina) [32], and triple-negative (TNBC) EO771 cells were kindly provided by Dr. Andrea Randi (Departmento de Bioquímica Humana, Facultad de Medicina, Universidad de Buenos Aires, Buenos Aires, Argentina). Human Luminal MCF7 and TNBC MDA-MB-231 and MDA-MB-468 cell lines were kindly provided by Dr. Natalia Rubinstein (Facultad de Ciencias Exactas y Naturales, Universidad de Buenos Aires, Buenos Aires, Argentina). For in vitro experiments, 50 µM of P60 or P301 peptides were used. Dose selection for cisplatin in all cell lines can be found in Supplementary Figure S1. 

### 2.3. Foxp3 Expression

LM3 cells were incubated for 24 h with 50 μM of rapamycin (Cayman Chemical Company, Ann Arbor, MI, USA, Cat# 53123-88-9), 50 μM of indomethacin (Química Montpellier, Buenos Aires, Aregentina), or 10 ng/mL of TGF-β (Cayman Chemical Company, Cat# 6164552UG). Mice tumor cells were incubated with two concentrations of cisplatin (2 and 20 µM) for 48 h. The cells were harvested with 0.025% trypsin–EDTA, washed with cold PBS, and prepared for flow cytometry analysis (see Supplementary Data).

Human MDA-MB-231 and MDA-MB-468 BRCA cells were seeded on coverslips in 24-well plates and incubated with or without cisplatin (40 and 4 µM, respectively) for 48 h. Foxp3 expression was assessed by immunocytochemistry and analyzed by fluorescence microscopy (see Supplementary Data).

### 2.4. Synthesis of P60

The therapeutic peptide P60 (RDFQSFRKMWPFFAM) and control peptide P301 (MKMFFDAFPQRRSWF) were synthesized by the solid-phase method of Merrifield using the fluorenylmethyloxycarbonyl alternative, as previously described [15]. The purity of peptides was 90%, as assessed by HPLC.

### 2.5. BrdU Cell Proliferation Assay

Cell proliferation was evaluated by incorporation of bromodeoxyuridine/5-bromo-2′-deoxyuridine (BrdU; SigmaAldrich, Roche, Darmstadt, Germany #Cat. 11647229001). Absorbance was determined using a 96-well plate spectrophotometer (Bio-Rad, Hercules, CA, USA, Model 550) at 490 nm as previously described [22,33,34].

### 2.6. Cell Viability

Cell viability was evaluated using the 3-(4,5-dimethylthiazol-2-yl)-2,5-diphenyltetrazolium bromide (MTT) assay (Invitrogen, Thermo Fisher Scientific, Waltham, MA, USA) as previously described [22,33,34].

### 2.7. Clonogenic Assay

LM3 cells were incubated with P60 or P301 (50 μM) for 1 h and then treated with cisplatin for 72 h. The cells were harvested with 0.025% trypsin–EDTA and seeded in plates of 6 wells at a density of 1000 cells/well. Ten days later, the cells were fixed in methanol for 10 min at −20 °C and stained with Giemsa stain. The number of colonies containing a minimum of 50 cells (colony forming unit, CFU) were counted using a binocular stereomicroscope and the clonogenic fraction was calculated based on the number of cells seeded relative to the number of clones formed.

### 2.8. Wound Assay

Cells were incubated in 24-well plates for 24 h with P60 or P301 (50 μM) or with conditioned medium (CM) from cells previously incubated with P60 or P301 for 48 h. Then, a wound was made in the wells with cells in confluence with a micropipette tip. The wells were washed with PBS and incubated again with the peptides or CM in complete medium without serum. Finally, the cells were photographed at different times and the wound area was measured in the images using ImageJ software. 

### 2.9. Zymography Assay

Cells were incubated with P60 or P301 (50 μM) alone or in the presence of cisplatin in the case of MDA-MB-231 cells for 48 h, and the gelatinocytic activity of metalloproteinases (MMP) was determined in their culture media by zymography. For this, 3 μL of medium was loaded into a gel of 10% acrylamide with 0.2% gelatin and run at 120 V. Next, the gels were washed with 50 mM Tris-HCl pH 7.5 in 2.5% Triton X-100 for 45 min, followed by another wash of 45 min in the same solution with 5 mM CaCl_2_ and 1 μM ZnCl_2_. The gels were then incubated for 24 h at 37 °C with a solution of 50 mM Tris-HCl with 10 mM CaCl_2_ and 200 mM NaCl at pH 7,5. Finally, the gels were stained with 0.5% Coomasie brilliant blue R-250 and faded with bleaching solution (25% *v*/*v* isopropanol–10% *v*/*v* acetic acid). The enzymatic activity, visualized as clear bands against a blue background, was analyzed by densitometry using ImageJ software. The zymographic activity was expressed as a percentage in relation to a standard internal sample saturating at a density of 50%. Data for different gels were normalized using internal control samples.

### 2.10. Propidium Iodide Exclusion Assay

A total of 60,000 cells were seeded per well in 24-well plates and treated according to the established conditions and incubation times. For sample preparation, independent tubes were used, and supernatants and cells previously detached with 0.025% trypsin–EDTA were collected. The samples were centrifuged for 5 min at 1500 rpm and the supernatant was discarded. For the preparation of the propidium iodide (PI) stock solution, 1 mg of PI was dissolved in 1 mL of distilled water, then the working solution was prepared using 1 µL of the stock solution in 100 µL of PBS. The cells were resuspended in 200 µL of the working solution and immediately analyzed by flow cytometry. Dead cells were identified by emitting fluorescence upon excitation at 488 nm.

### 2.11. Fluorometric Caspase-3 Activity Test

Caspase-3 activity in total BRCA cells lysates was evaluated with the EnzChek^®^ Caspase-3 Test Kit No. 2 (Invitrogen, Thermo Fisher, Cat# E-13184) using rhodamine acid amide 110 bis-(NCBZ-L-aspartyl-Lglutamyl-L-valyl-L-aspartic) (Z-DEVD–R110) according to the manufacturer’s instructions. Briefly, the cells were incubated with the P60 peptide and its P301 control with or without cisplatin (5 uM). After 48 h, the cells were collected and total cell lysates prepared. Next, samples of the cell lysates were seeded into 96-well plates and mixed with a reaction buffer including caspase-3 substrate (25 μM Z-DEVD-R110), and the plates were incubated for 1 h at room temperature protected from light. The caspase-3 activity of the cell extracts was determined by a fluorescence microplate reader (Biotek Synergy H1, Agilent, Santa Clara, CA, USA) using excitation/emission wavelengths of 496/520 nm. Sample readings were calculated by subtracting the absorbance of the blank sample (without extract).

### 2.12. Adenoviral Vectors

We constructed a non-replicative human adenovirus serotype 5 vector (AdV) encoding the red fluorescent *dTomato* reporter gene or the penetrating peptide P60 followed by a sequence internal ribosome entry site (IRES) and the *dTomato* gene, under the control of the CMV immediate–early promoter, using the ViraPower™ Adenoviral Expression System (Invitrogen, Thermo Fisher Scientific) and following the manufacturer’s recommendations as previously described [35]. Briefly, the heterologous nucleotide sequences, including the dTomato-SV40 polyA (1030 bp) or P60-IRES-SV40 polyA (1680 pb), were amplified by PCR using specific primer oligonucleotides and the pl.dT or pl.P60 plasmids as templates (synthesized by Macrogen, Seoul, Republic of Korea). The PCR amplification products were first cloned into the pGEMT-Easy T vector (Promega, Madison, WI, USA) and then subcloned into the BamHI and HindIII sites of the polylinker from the entry vector pENTR4 (Invitrogen, Thermo Fisher Scientific). Then, in vitro homologous recombination reactions between the att1 and att2 sites of the entry vectors pENTR4.dTomato or pENT4.P60 and the destination adenoviral vector pAdV-CMV/V5-DEST (Invitrogen, Thermo Fisher Scientific) allowed us to obtain the plasmids pAdV-CMVdTomato and pAdV-CMVP60, respectively. These constructs contained the heterologous sequences downstream of the CMV promoter and replaced the coding region for the E1 and E3 proteins in the adenovirus genomes. The identity of the pAdV-CMV-dTomato and pAdV-CMVP60 were confirmed by nucleotide sequencing (Macrogen). To obtain the recombinant adenoviral vectors (AdV.dT), the plasmids pAdVCMV-dTomato and pAdV-CMVP60 were digested with the restriction enzyme PacI (which allows exposing the ITRs regions) and transfected into HEK293A cultures. This cell line constitutively expressed the product of the *E1* viral gene, allowing the formation of the infective viral particles (Ad.dT and Ad.P60). Viral stocks were harvested after the appearance of the cytopathic effect and amplified by passages in fresh monolayers. The insertion of the foreign sequences in the recombinant AdVs was confirmed by PCR with specific oligonucleotides using total DNA extracted from infected cells as template. Similarly, the expression of the reporter gene was evidenced by microscopic observation of the red fluorescence emitted in infected cells and exposed to ultraviolet light. Titers of recombinant adenoviruses were determined using a 96-well tissue culture plate seeded with 3 × 10^3^ 293 cells. Cells were incubated with a series of 10- and 2-fold dilutions of the recombinant adenoviruses in triplicate. After 8 days, we read the plate for the highest dilution in which all three wells contained plaques and calculated the PFU/mL following Southgate et. al.’s description [36].

### 2.13. Animals

Adult female *BALB/c* and *C57Bl/6* (6–8 weeks old) mice were purchased at the vivarium of Facultad de Ciencias Veterinarias, Universidad Nacional de La Plata. *C57Bl/6J* transgenic Foxp3-GFP mice (*B6.Cg-Foxp3^tm2Tch^/J*) were kindly provided by Dr. Eva Acosta, Facultad de Ciencias Químicas, Universidad Nacional de Córdoba. Mice were maintained under controlled conditions of light (12 h light–dark cycles) and temperature (20–25 °C). Mice were fed with standard lab chow and water ad libitum and all efforts were made to minimize distress. 

### 2.14. In Vivo Experimental Breast Cancer Models

*BALB/c* mice or *C57Bl/6* mice were inoculated s.c. into the flank with 2 × 10^6^ tumor LM3 or EO771 cells, respectively. Tumor size was measured 3 times per week using a caliper. The tumor volume was calculated with the following formula: (width^2^ × length)/2. When tumors reached 500 mm^3^, mice were inoculated i.t. with 6.3 × 10^7^ PFU of Ad.dT or Ad.P60 every 3 days for a total of 3 injections. Mice were monitored daily, and when one mouse presented the first signs of distress, the mice were euthanized by cervical dislocation. Then, lungs were dissected and fixed with Bouin’s fixative solution (71% picric acid (saturated), 24% formaldehyde (37–40%), and 5% glacial acetic acid) and spontaneous metastases were counted under a binocular stereoscopic microscope.

A group of EO771-tumor-bearing *C57Bl/6* mice was euthanized 3 days after Adv inoculation by terminal perfusion under deep anesthesia with Tyrode’s solution (NaCl 132 mM, CaCl_2_ 1.8 mM, NaH_2_PO_4_ 0.32 mM, glucose 5.56 mM, NaHCO_3_ 11.6 mM, and KCl 2.68 mM) followed by 4% PFA. The presence of cells expressing the reporter protein dTomato was assessed in tumor sections by fluorescent microscopy (see Supplementary Data).

A group of EO771-tumor-bearing *C57Bl/6J* transgenic mice was euthanized 8 days after Adv inoculation and the content of lymphocytes was assessed in spleen and tumor as previously described [22,37,38] (see Supplementary Data).

All animal experimentation was performed under the guidance of the NIH and was approved by the Institutional Committee for the Care and Use of Laboratory Animals (CICUAL), School of Medicine, University of Buenos Aires; Res. (CD) No. 2071/15.

### 2.15. Statistical Analysis

Data were plotted and analyzed using GraphPad Prism version 8.00 software. All data were tested for normality using the Kolmogorov–Smirnov test and the Shapiro–Wilk test before performing parametric statistical tests. Continuous variables were compared using either Student’s *t*-test, the one-way analysis of variance test (ANOVA), or two-way ANOVA. Correlations between continuous variables were assessed using Spearman’s correlation analysis. Differences between groups were considered significant when *p* < 0.05. All experiments were performed at least twice. 

## 3. Results

### 3.1. Foxp3 Mediates Chemoresistance in Breast Cancer Cells

**Figure 1:**
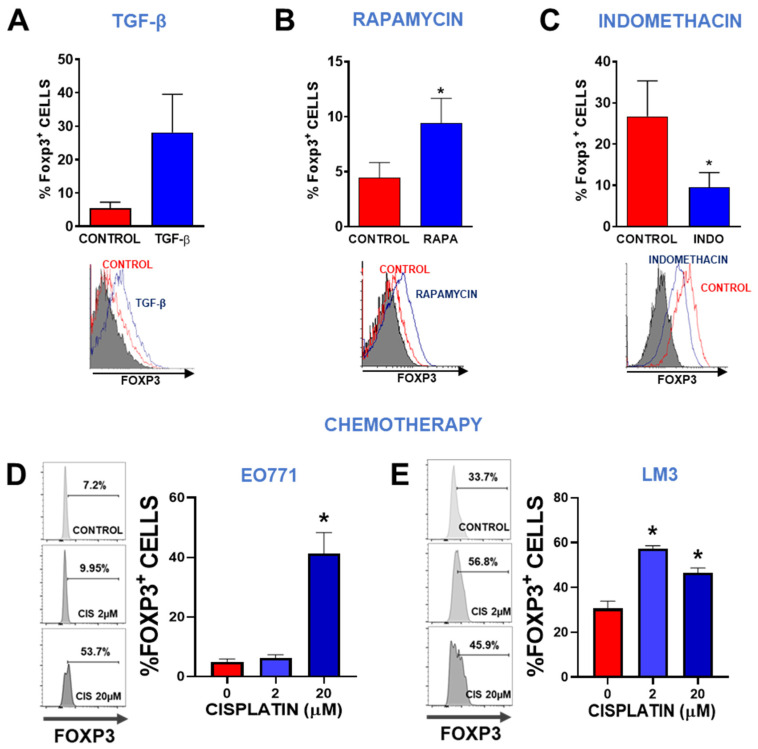
Regulation of Foxp3 expression. Foxp3 expression was assessed by flow cytometry in LM3 cells incubated for 24 h in the presence of 10 ng/mL of recombinant TGF-β (**A**), 50 μM of the mTOR pathway inhibitor rapamycin (RAPA) (**B**), or 50 μM of the COX-2 inhibitor indomethacin (INDO) (**C**). Representative histograms are shown. The histograms of cells incubated with isotype controls are shown in gray. * *p* < 0.05 vs control (Student’s *t*-test). (**D**) TNBC subtype EO771 breast cancer cells and (**E**) HER2+ LM3 breast cancer cells were incubated with 2 or 20 μM cisplatin for 48 h and then Foxp3 expression was evaluated by flow cytometry. Representative histograms are shown. * *p* < 0.05, ANOVA.

Considering that we and others had previously shown that human and murine breast cancer (BRCA) cells express Foxp3 [19,20,21,22], we hypothesized that the mechanism involved in the regulation of Foxp3 expression in Tregs was similar in BRCA cells. We have also reported that Foxp3 mediates BRCA cell survival, proliferation, and IL-10 secretion, effects that are similar to those observed in Tregs [22]. Thus, we evaluated the effect of TGF-β, the mTOR inhibitor rapamycin, and the COX inhibitor indomethacin, which have been shown to regulate Foxp3 expression in Tregs [39]. By flow cytometry, we found that incubation with recombinant TGF-β (Figure 1A) and rapamycin (Figure 1B) increased Foxp3 expression in LM3 cells, whereas indomethacin downregulated it (Figure 1C). These results suggest that the mechanisms that regulate Foxp3 expression in BRCA cells are similar to those described in Tregs [40]. We next evaluated whether chemotherapy affects the expression of Foxp3 in EO771 and LM3 murine BRCA cells. Flow cytometric analysis of TNBC EO771 cells revealed that, although only 10% of these cells constitutively expressed Foxp3, incubation with 20 µM cisplatin raised this percentage to near 50% (Figure 1D). Similarly, we observed that approximately one-third of HER2^+^ LM3 cells expressed Foxp3 at baseline, as previously reported [22], and that cisplatin significantly upregulated its expression, even at low concentrations (2 µM) (Figure 1E).

**Figure 2:**
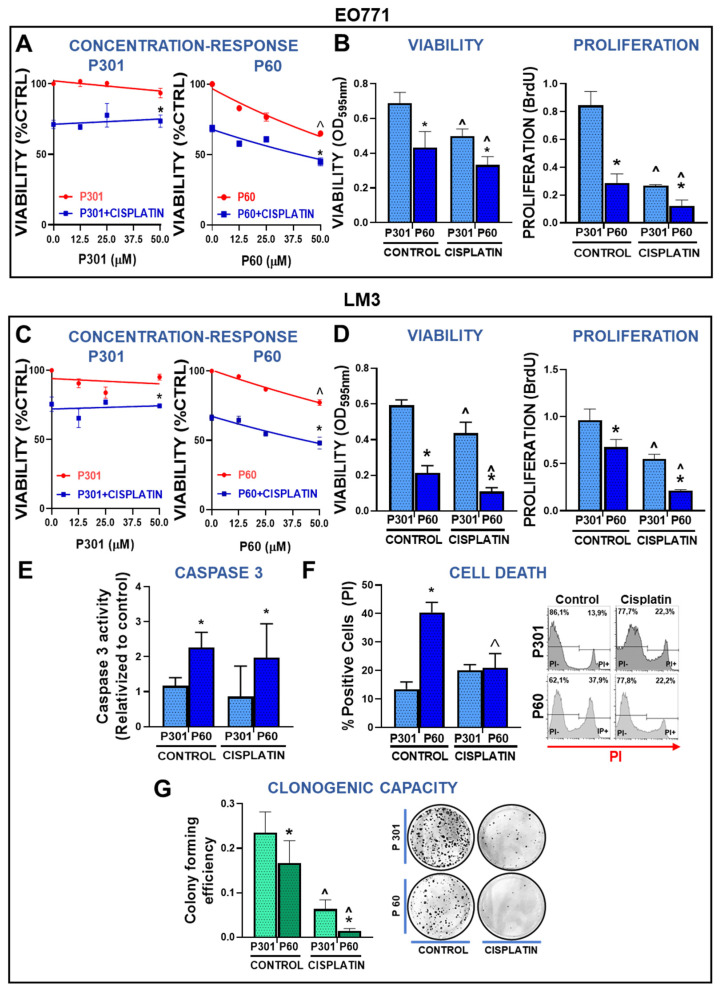
Foxp3 tumor intrinsic effects in breast cancer cells: chemoresistance. (**A**) TNBC EO771 and (**C**) HER2+ LM3 cells were incubated with peptide P60 or P301 at concentrations of 12.5, 25, or 50 μM in the presence or absence of 5 μM cisplatin for 72 h. Cell viability was determined by MTT assay. Viability values were expressed as a percentage of control without any treatment. * p < 0.05 vs. *respective control*, ˆ *p < 0.05* vs. *peptide concentrations, 2-way ANOVA*. (**B**) EO771 and (**D**) LM3 cells were incubated with P301 or P60 (50 μM) in combination with 5 μM cisplatin and viability (by MTT assay) and cell proliferation (by BrdU incorporation) were evaluated 72 h later. * p < 0.05 vs. *P301*; *^ p < 0.05* vs. *respective control without cisplatin, 2-way ANOVA.* (**E**) The activity of caspase 3 was measured by fluorescence intensity in LM3 cells incubated for 48 h with P301 or P60 (50 μM) and cisplatin (5 μM). (**F**) LM3 cells were incubated for 48 h with P301 or P60 (50 μM) and cisplatin (5 μM), and the percentage of dead cells was determined by propidium iodide incorporation measured by flow cytometry. Representative histograms are shown and depict the percentage of dead cells. * *p < 0.05* vs. *P301*; *^ p < 0.05* vs. *respective control without cisplatin, 2-way ANOVA*. (**G**) The clonogenic capacity of LM3 cells was assessed 72 h after the incubation with P301 or P60 (50 μM) in the presence or absence of cisplatin (2 μM). * p < 0.05 vs. *P301*; *^ p < 0.05* vs. *respective control without cisplatin, 2-way ANOVA*.

We had previously observed that the blockade of Foxp3 nuclear translocation with the CPP P60 decreased the viability and proliferation of BRCA cells [22]. Thus, we evaluated whether Foxp3 blockade affects the response of these cells to chemotherapy. To establish an optimal concentration for P60, we performed a concentration–response curve of P60 and its control peptide P301 [15] in the presence of cisplatin in LM3 and EO771 cells. We found that P60 significantly reduced cell viability and increased sensitivity to cisplatin in a concentration-dependent manner, whereas the control peptide did not exert any effect (Figure 2A,C). We chose 50 µM as working concentration for the experiments that followed. We assessed the viability and proliferation of EO771 and LM3 cells treated with cisplatin in the presence of P60 or its control peptide P301. P60 not only decreased the viability and proliferation of both EO771 (Figure 2B) and LM3 cells (Figure 2D) but it boosted the cytotoxic and antiproliferative effect of cisplatin (Figure 2B,D). 

To further study the effect of P60 in BRCA cells, we evaluated whether this peptide induces cell death in these cells. We evaluated caspase 3 activity in LM3 cells incubated with P301 or P60 in the presence of cisplatin for 48 h. P60 significantly upregulated caspase 3 activity in comparison with P301 (Figure 2E). However, we failed to detect the stimulatory effect of cisplatin on caspase 3 activity at this time of incubation. We also evaluated cell death by the propidium iodide (PI) exclusion assay. We found that blockade of Foxp3 nuclear translocation significantly increased cell death (Figure 2F). However, no effect of cisplatin alone or in combination with P60 on cell death was detected at the time of evaluation (Figure 2F). It is possible that due to the potent cytotoxic effect of cisplatin+P60, the peak of PI positive cells was already surpassed by the time of evaluation. 

We next evaluated whether P60 affects the ability of cisplatin to induce mitotic catastrophe in BRCA cells using the clonogenic assay. LM3 cells were treated with cisplatin and those that were alive at 72 h were seeded at low density in order to evaluate the formation of individual clones. Treatment with P60 alone reduced the clonogenic capacity of LM3 cells (Figure 2G). Treatment with cisplatin also inhibited the clonogenic response of LM3 cells, an effect that was significantly improved by the blockade of Foxp3 using P60 (Figure 2G).

### 3.2. Foxp3 Facilitates Migration of Breast Cancer Cells

**Figure 3:**
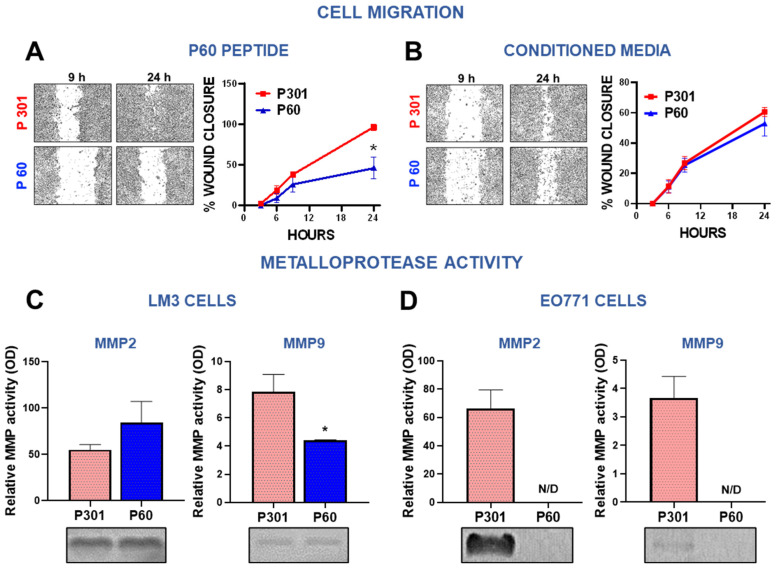
Foxp3 tumor intrinsic effects in breast cancer cells: invasion. (**A**,**B**) LM3 breast cancer cells were incubated for 24 h with P301 or P60 (50 μM) (**A**) or with the conditioned medium of cells previously incubated for 48 h with these peptides (**B**). The wound assay was performed and the migratory capacity of the cells evaluated. Representative microphotographs of the wound at different time points are shown. * p < 0.05, non-linear regression analysis. (**C**,**D**) The MMP-2 and MMP-9 activity was assessed by zymography in incubation media of EO771 cells (**C**) and LM3 cells (**D**) treated with P301 or P60 (50 μM) for 48 h. The gels were stained with Coomassie blue, and the bands were analyzed by densitometry with the ImageJ software in triplicate. Zymographic activity was expressed as a percentage relative to a standard internal sample saturating at a density of 50%. Representative bands for each group are shown.

Since our previous results indicated that treatment with P60 reduces the development of lung metastases in experimental BRCA [22], we evaluated whether Foxp3 affects the migratory capacity of LM3 cells using the wound assay. LM3 cells were incubated with P60, its control peptide P301, or with conditioned media (CM) from cells treated with these peptides. Direct treatment of LM3 with P60 significantly delayed wound closure (Figure 3A), suggesting that Foxp3 facilitates migration of these cells. However, incubation with CM from P60-treated cells did not affect cell migration (Figure 3B). To further understand the role of Foxp3 in BRCA cell invasion, we evaluated the activity of matrix metalloproteases (MMPs) required to promote tumor invasion and metastasis [41]. Using a zymography assay [42] we measured the expression of MMP-2 and MMP-9 in the CM of EO771 and LM3 BRCA cells incubated with P60 or P301 (Supplementary Figures S2 and S3). Both cell lines exhibited a higher baseline content of active MMP-2 than MMP-9 (Figure 3C,D). In LM3 cells, we observed that blockade of Foxp3 significantly reduced the content of active MMP-2 without affecting the already low levels of active MMP-9 (Figure 3C). On the other hand, treatment with P60 resulted in a complete inhibition of MMP-2 and MMP-9 secretion in EO771 cells, being below detection threshold when Foxp3 was inhibited (Figure 3D). These observations suggest that Foxp3 may facilitate BRCA invasion by promoting cell migration and stimulating the secretion of matrix remodeling enzymes. 

### 3.3. Foxp3 Does Not Affect the Angiogenic Capacity of Breast Cancer Cells

We also evaluated if Foxp3 blockade affects the capacity of BRCA cells to secrete proangiogenic factors using the wound assay in EA.hy.926 endothelial cells. The incubation of EA.hy.926 cells with CM or with P60 did not affect the migratory capacity of EA.Hy.926 cells (Supplementary Figure S4), suggesting that Foxp3 does not participate in the modulation of the tumor angiogenic process.

### 3.4. Foxp3 Tumor Intrinsic Effects in Human Breast Cancer Cells

**Figure 4:**
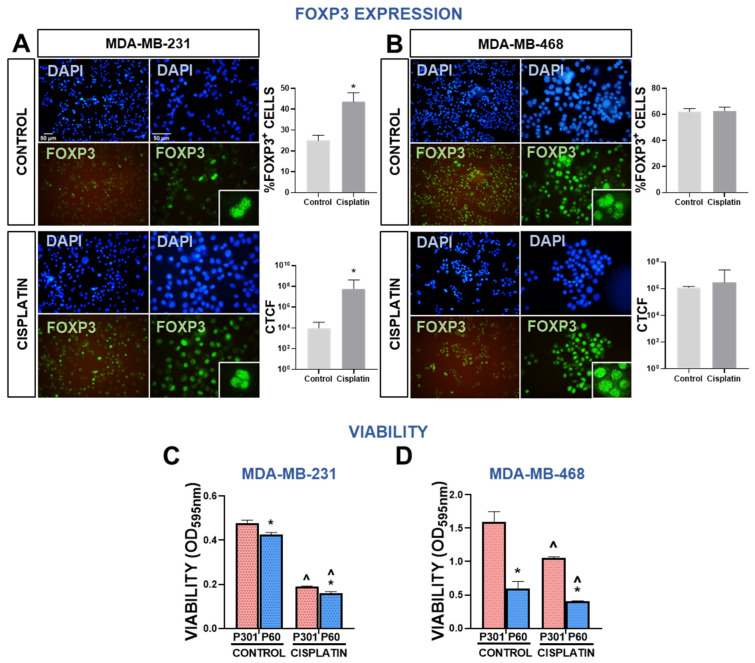
Foxp3 expression and response to chemotherapy in human breast cancer cells. (**A**,**B**) Expression of Foxp3 in MDA-MB-231 (**A**) and MDA-MB-468 cells (**B**) after incubation with 40 μM or 4 μM cisplatin, respectively, was assessed by indirect immunofluorescence. Representative microphotographs show Foxp3 expression (green). Nuclei were counterstained with DAPI (blue). The percentage of cells with high intensity nuclear Foxp3 and intensity of fluorescence, expressed as corrected total cell fluorescence (CTCF), was assessed using ImageJ software. * p < 0.05 (Student’s t test). (**C**,**D**) Chemosensitivity and viability (by MTT) were evaluated in MDA-MB-231 (**C**) and MDA-MB-468 (**D**) human TNBC cells incubated for 72 h with P301 or P60 (50 μM) in the presence or absence of cisplatin (5 μM and 2 μM, respectively). * p < *0.05* vs. *P301; ^ p* < *0.05* vs. *respective control without cisplatin, 2-way ANOVA*.

We evaluated the tumor intrinsic effects of Foxp3 in MDA-MB-231 and MDA-MB-468 human TNBC cells. First, we assessed the Foxp3 expression in presence or absence of cisplatin. Tallying with previous reports [22], basal expression of Foxp3 in MDA-MB-231 was relatively lower than that in MDA-MB-468 cells (Figure 4A,B). However, incubation with cisplatin significantly upregulated Foxp3 expression in MDA-MB-231 cells, which was detected in almost 50% of the cells **(**Figure 4A). Cisplatin did not affect the already high expression of Foxp3 in MDA-MB-468 cells. We next evaluated the effect of Foxp3 blockade on cell viability and chemosensitivity. P60 (50 μM) decreased the viability of MDA-MB-231 cells and slightly improved the cytotoxic effects of cisplatin (Figure 4C). These effects were even more pronounced in MDA-MB-468 cells (Figure 4D). 

**Figure 5:**
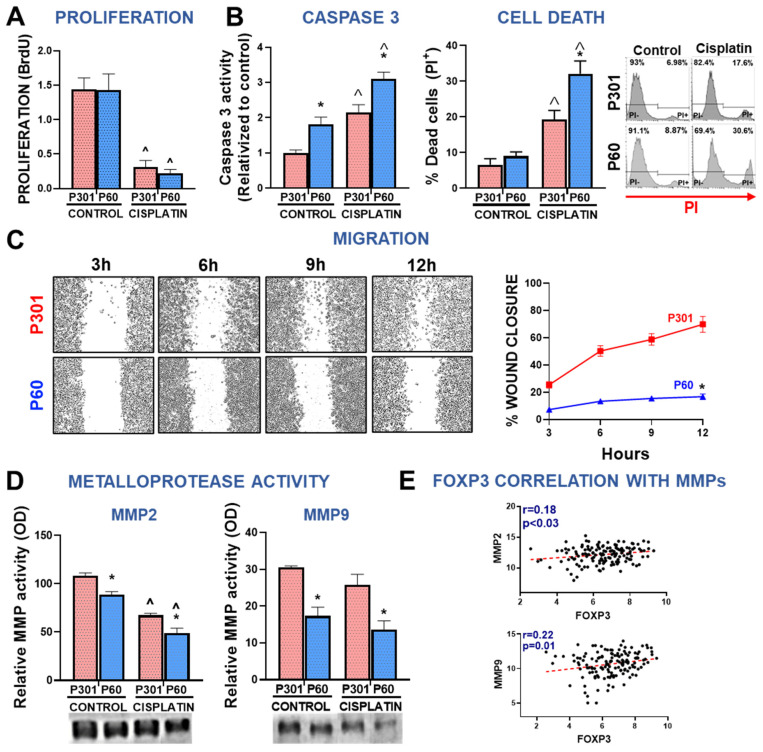
Foxp3 tumor intrinsic effects in human breast cancer cells: chemoresistance and invasion. Proliferation (**A**), apoptosis (**B**), migration (**C**), and secretion of active MMPs (**D**) were evaluated in MDA-MB-231 cells incubated for 72 h with P301 or P60 (50 μM) in the presence or absence of cisplatin 5 μM. * p < 0.05 vs. *P301; ^ p < 0.05* vs. *respective control without cisplatin;* (**A**,**B**,**D**), *2-way ANOVA;* (**C**)*, Non-linear regression analysis.* (**E**) Spearman correlation r values between FOXP3 levels and MMP mRNA levels from TNBC biopsies from TCGA BRCA database.

Since cisplatin increased Foxp3 levels in MDA-MB-231 cells, we further evaluated other Foxp3 intrinsic effects in these cells. Foxp3 blockade using P60 peptide did not affect the proliferative capacity of MDA-MB-231 cells even in combination with cisplatin (Figure 5A). To evaluate the effect of P60 on the apoptotic response of BRCA cells, we evaluated caspase 3 activity and the rate of cell death by PI exclusion in MDA-MB-231 cells. Foxp3 blockade significantly upregulated caspase 3 activity per se and boosted cisplatin-induced cell death (Figure 5B). We next evaluated the effect of P60 on the migratory capacity of MDA-MB-231 cells. We found that P60 exerted a robust inhibition of the migration of these cells (Figure 5C). Conversely, when we evaluated the effect of P60 on the migration capacity of the luminal subtype cell line MCF-7, we did not observe differences compared with P301 (Supplementary Figure S5). Along with these results, we observed that Foxp3 blockade significantly reduced the secretion of active MMP-2 and MMP-9 in MDA-MB-231 cells (Figure 5D) (Supplementary Figure S6). Interestingly, cisplatin also inhibited secretion of these MMPs and P60 boosted this effect. Furthermore, when we analyzed the transcriptomic data from TNBC subtype BRCA biopsies deposited in TCGA, we found that tumor expression of Foxp3 positively correlated with the expression of MMP-2 and MMP-9 (Figure 5E). These results indicate that Foxp3 exerts protumoral intrinsic effects in BRCA cells that involve not only sustaining cell survival and resistance to apoptosis but also accelerating their migration, suggesting that this transcription factor could facilitate BRCA tumor progression.

### 3.5. Development and Characterization of Ad.P60

**Figure 6:**
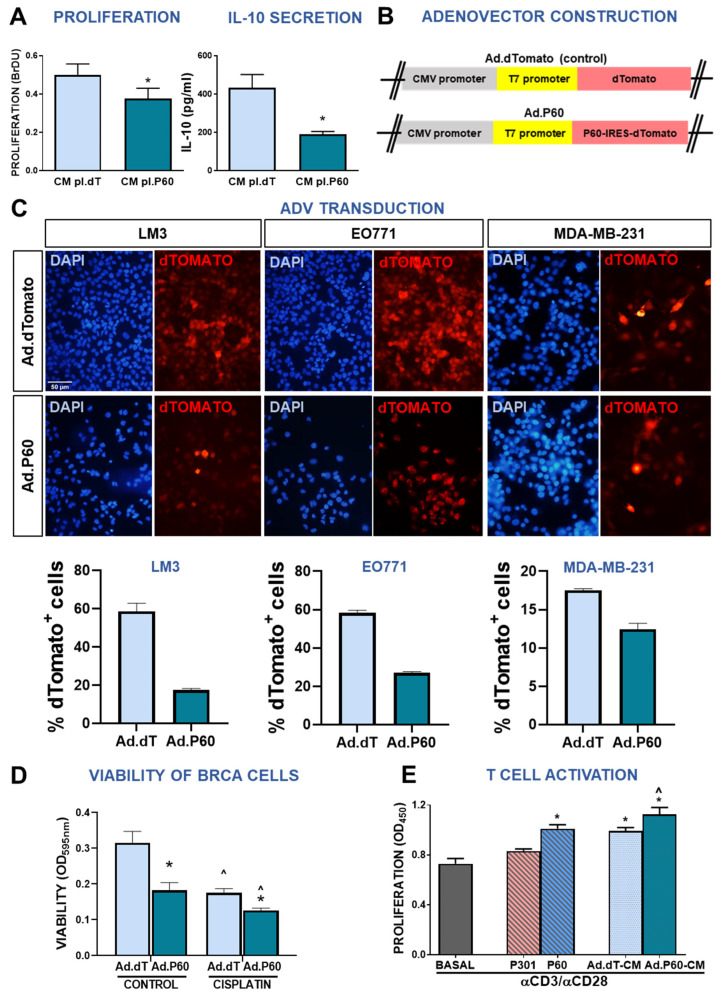
Development and characterization of an adenoviral vector encoding Foxp3 inhibitory peptide P60. (**A**) LM3 cells were incubated for 24 h with conditioned medium from 4T1 cells transfected with a plasmid encoding P60 (pl.P60) or a control plasmid (pl.dT) for 24 h. Proliferation (left panel) and IL-10 secretion (right panel) were evaluated. * *p* < 0.05 (Student’s *t* test). (**B**) Schematic representation of the vector encoding P60 (Ad.P60) or its control (Ad.dT). (**C**) Murine LM3 and EO771 cells and human MDA-MB-231 breast cancer cells were transduced with Ad.dT or Ad.P60 for 48 h at multiplicity of infection (MOI) of 200. Transduction efficiency was assessed by detection of cells expressing the reporter gene dTomato using fluorescence microscopy, and the percentage of dTomato+ cells was measured using ImageJ software. Nuclei were stained with DAPI. (**D**) The direct effect of Ad.dT and Ad.P60 was evaluated in EO771 cells transduced with the vectors at a MOI of 200 in combination with cisplatin (5 μM). After 72 h of transduction, viability was assessed by MTT. * *p* < 0.05 vs. control, ˆ *p* < 0.05 vs. respective control without cisplatin; 2-way ANOVA. (**E**) Murine splenocytes were incubated in the presence of P301 or P60 or with conditioned medium (CM) from EO771 transduced cells and activated with αCD3 and αCD28 antibodies. T cell proliferation was assessed by BrDU incorporation. * *p* < 0.05 vs. basal control, ˆ *p* < 0.05 vs. peptide P60 2-way ANOVA.

To improve the local availability of P60 and avoid the systemic distribution of the peptide in vivo, we developed gene therapy vectors that encode for this peptide. We first generated a plasmid encoding for the sequence of P60 linked to the reporter gene for the red fluorescent protein dTomato by an IRES sequence (pl.P60), followed by a polyA sequence under the control of the CMV promoter. We also constructed a control vector that expresses the reporter gene (pl.dT). To assess the capacity of pl.P60-transfected cells to express the P60 peptide, LM3 cells were incubated with CM from transfected 4T1 cells that express low levels of Foxp3. The CM of pl.P60-transfected 4T1 cells significantly reduced LM3 cell proliferation and IL-10 secretion compared with control pl.dT CM (Figure 6A). 

Based on our results, we used these plasmids to develop the non-replicative type 5 recombinant adenoviral vectors (Advs) Ad.P60 and its control Ad.dT (Figure 6B). We evaluated the transduction efficiency of Ad.P60 by immunofluorescence in the murine BRCA cell lines LM3 and EO771 and in the human cell line MDA-MB-231, incubating these cells with Ad.dT or Ad.P60 at a multiplicity of infection (MOI) of 200 for 48 h (Figure 6C). Although both vectors readily transduced all of the cells evaluated, the percentage of positive cells was lower when they were transduced with Ad.P60. We next evaluated whether Ad.P60 transduction led to the expression of functional P60 peptide. To this end, we studied the effect of Ad.P60 on the viability and chemoresistance of EO771 cells incubated with Ad.P60 at an MOI of 200 for 72 h. Transduction with Ad.P60 significantly reduced the viability of BRCA cells per se and increased their response to cisplatin (Figure 6D), which is consistent with our findings using the peptidic formulation of P60 (Figure 2). 

We then evaluated whether the CM from cells transduced with the Adv exerted immunomodulatory effects. Splenocytes are a heterogeneous population of lymphocytes with approximately 3% of Tregs [38,43]. Splenocytes are activated using antibodies specific to CD3 and CD28 co-activating molecules, which induce the proliferation of T lymphocytes [44]. Tregs present in the co-culture reduce the proliferative capacity of helper and effector T cells and P60 has been shown to inhibit Treg-mediated negative modulation of effector T lymphocytes [15]. We incubated splenocytes with P60 peptide as a positive control and observed a significant increase in the response of T lymphocytes to αCD3/αCD28-induced activation (Figure 6E). The same effect was observed when we incubated splenocytes with the CM of EO771 cells transduced with Ad.P60, which exhibited an augmented proliferative response (Figure 6E). These results indicate that transduction of EO771 cells with Ad.P60 not only inhibits the intrinsic protumoral effects of Foxp3 in these cells but also favors the proliferation of T lymphocytes through a bystander effect.

### 3.6. Study of the In Vivo Therapeutic Efficacy of Ad.P60 in Experimental Breast Cancer Models

**Figure 7:**
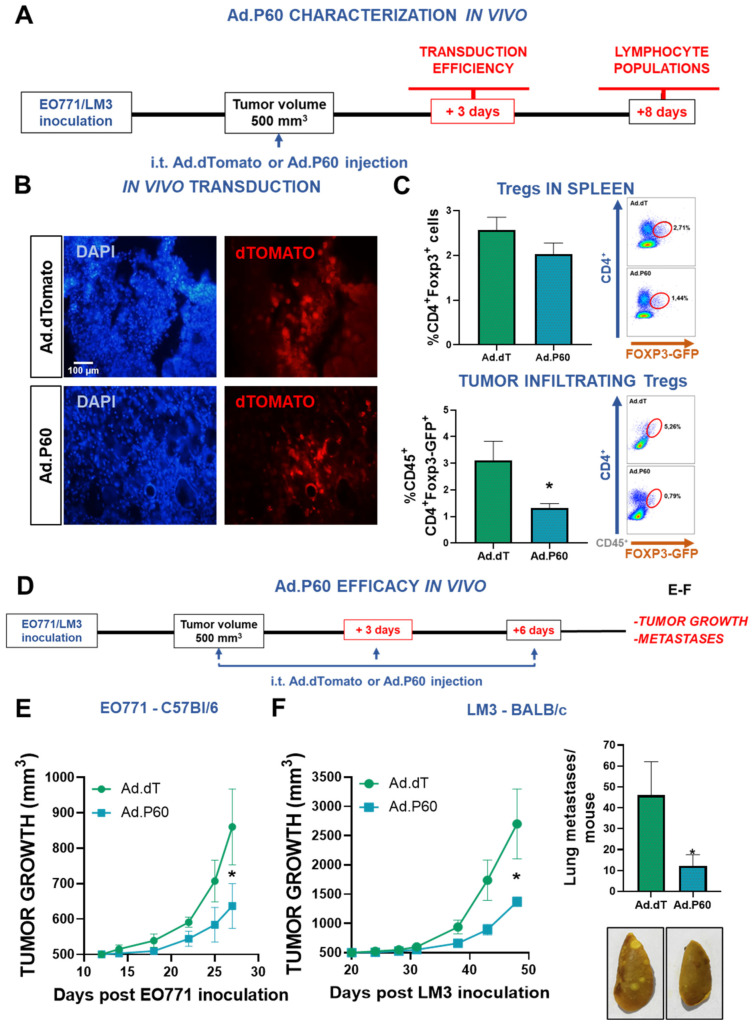
Efficacy of Ad.P60 in experimental breast cancer models. (**A**) *C57Bl/6* mice were subcutaneously injected with 200,000 EO771 cells. When tumor volume reached 500 mm^3^, mice were treated i.t. by inoculating 6.3 × 10^7^ PFU of Ad.dT or Ad.P60. (**B**) After 3 days, transduction efficiency was evaluated in tumor cryostat slices by detection of the reporter protein dTomato by fluorescence microscopy. The nuclei of the cells were stained with DAPI. (**C**) A total of 8 days after inoculation, spleen and tumor-infiltrating Treg populations were evaluated in *C57Bl/6J* mice transgenic for Foxp3-GFP. Immune populations were analyzed by flow cytometry, studying the presence of CD4^+^ Foxp3-GFP+ T cells (*Student’s t test*; * *p* < 0.05). (**D**) Tumor growth was evaluated in *C57Bl/6* mice bearing syngeneic EO771 tumors (**E**) and *BALB/c* mice bearing LM3 tumors (**F**) that were treated with 3 i.t. injections of Ad.dT or Ad.P60. * p < 0.05; multiple regression analysis. (**F**) Total number of spontaneous lung metastases in each mouse bearing LM3 tumors. Representative images of lungs are shown, * p < 0.05 (Student’s t test).

We evaluated the efficacy of Ad.P60 in vivo following the scheme depicted in Figure 7A. The transduction efficiency was evaluated 3 d after intratumoral (i.t.) injection with 6.3 × 10^7^ PFU of Ad.dT or Ad.P60 in subcutaneous EO771 tumors established in *C57Bl/6* mice. Cells expressing the fluorescent reporter gene were readily detected in tumors injected with Ad.dT or Ad.P60, demonstrating that these vectors are capable of transducing breast tumor cells in vivo (Figure 7B). Our previous findings indicated that systemic administration of P60 reduces vaccine-induced expansion of Tregs in established breast tumors and the spleen [22]. To assess whether i.t. inoculation of Ad.P60 affected the content of Tregs in spleen and tumor, we used Foxp3-EGFP reporter *C57Bl/6J* transgenic mice that were engineered to express an *IRESEGFP* sequence inserted at the 3′ end of the *Foxp3* gene (*B6.Cg-Foxp3^tm2Tch^/J*) [45,46]. A total of 7 d after Ad.dT or Ad.P60 i.t. injection, we did not observe differences in the content of the CD4^+^ and CD8^+^ populations in spleen or the tumor (Supplementary Figure S7). However, mice treated with Ad.P60 exhibited a significant lower number of tumor infiltrating Tregs (CD45^+^CD4^+^Foxp3-GFP^+^) than those administered with Ad.dT (Figure 7C). Interestingly, i.t. injection of Ad.P60 did not alter the content of Tregs (CD4^+^Foxp3-GFP^+^) in the spleen (Figure 7C). 

Later, we evaluated the therapeutic efficacy of Ad.P60 in *C57Bl/6* mice bearing EO771 tumors and *BALB/c* mice bearing LM3 tumors. When tumors reached a volume of 500 mm^3^, we started a treatment that consisted of i.t. injections with 6.3 × 10^7^ PFU of Ad.dT or Ad.P60 every 3 d for a total of 3 times and monitored tumor growth. We observed that local treatment with Ad.P60 significantly reduced tumor growth compared with that in mice treated with control vector Ad.dT in both tumor models (Figure 7E,F). Furthermore, treatment with Ad.P60 significantly reduced the development of spontaneous lung metastases in LM3 bearing *BALB/c* mice (Figure 7F).

## 4. Discussion

One of the main mechanisms of tumor evasion is the recruitment of Tregs, which exert suppressive effects on effector T lymphocytes [47]. An increase in tumor-infiltrating Tregs is associated with a worse prognosis in BRCA patients, making these cells an important therapeutic target [7,48]. The infiltration of Foxp3+ Tregs as well as the expression of Foxp3 in breast tumor cells, together with their subcellular localization, were proposed as prognostic markers for BRCA [19]. Although there is consensus on the tumor suppressive role of Foxp3 in the normal mammary gland [25], its involvement in the pathogenesis of BRCA remains controversial [26,27]. Here, we aimed to better understand the tumor intrinsic effects of Foxp3 in BRCA cells. 

Although the expression of Foxp3 has been reported in many tumor cell types, the regulation of its expression has been barely explored. TGF-β is a potent inducer of Foxp3 expression in Tregs [49,50,51] and has also been reported to control Foxp3 expression in pancreatic carcinoma cells [52]. We found that stimulation with TGF-β upregulated Foxp3 in BRCA cells. Since TGF-β is produced at high levels in the TME, it is likely that it favors the expression of Foxp3, not only in Tregs but also in tumor cells. Importantly, whereas Foxp3 upregulates TGF-β expression in Tregs by repressing its negative regulator [3], TGF-β also plays a central role in epithelial–mesenchymal transition (EMT) [53], and it is possible that a positive feedback loop between Foxp3 and TGF-β could facilitate EMT in BRCA. Rapamycin, an inhibitor of the mTOR pathway, is an immunosuppressive agent that has been used to prevent transplant rejection in humans [54,55] as it induces the expansion of highly suppressive Tregs [56,57]. Similarly, prostaglandin PGE2, which has been postulated to differentiate Tregs, increases Foxp3 expression in resting CD4+CD25+ cells [58]. The inhibition of the inducible enzyme cyclooxygenase, the major source for PGE2 production, with indomethacin was observed to decrease Foxp3 levels and thus reduced the levels of antigen-induced Tregs [59]. In our experiments, we observed that these regulatory mechanisms of Foxp3 expression in Tregs are conserved in BRCA cells, suggesting that the therapeutic interventions intended to inhibit Foxp3 expression in Tregs could also be useful to repress the expression of this transcription factor in BCRA cells. 

Cisplatin is a widely used therapeutic agent for the treatment of solid tumors. Despite its observed toxic side effects, [60] it is commonly used for the treatment of metastatic TNBC tumors that possess a deficient DNA repair system [61,62]. Although its systemic toxicity limits its use in oncologic patients, strategies that sensitize tumor cells to its cytotoxic effects could expand the use of this powerful chemotherapeutic drug. Our results show that cisplatin stimulates the expression of Foxp3 in BRCA cells. Considering that chemotherapy can also induce the expression of TGF-β [63] or the generation of reactive oxygen species [64], it is possible that these mechanisms participate in the regulation of Foxp3 induced by cisplatin in BRCA cells. Another possibility is that since Foxp3 expressing cells show enhanced resistance to cisplatin, these cells prevail after cisplatin treatment. Since blockade of Foxp3 function sensitized BRCA cells to the cytotoxic and antiproliferative effects of cisplatin, Foxp3 expression may be one of the mechanisms that mediates chemoresistance in these cells. 

The ability of tumor cells to invade tissues is critical for tumor progression. Considering that Foxp3 blockade using P60 inhibited the expression of MMPs in BRCA cells in vitro, it is possible that Foxp3 upregulates the expression and/or activity of MMPs. In fact, we found that the expression of Foxp3 was positively correlated with the expression of MMPs in GBM biopsies. Moreover, Foxp3 blockade in vivo reduced the metastatic potential of breast cancer cells. Our findings agree with previous reports that show that Foxp3 expression upregulates MMP-1 expression in hepatocellular carcinoma cancer cells, facilitating invasion and metastases [65]. Foxp3 was also associated with upregulation of MMP2 and MMP9 in cholangiocarcinoma cells and lung cancer cells, EMT, and metastasis [66,67]. Further, it was reported that cisplatin reduced MMP-2 activity in a dose and time-dependent manner in transformed thyroid cells [68]. We observed that a combination of cisplatin with Foxp3 blockade has a significant effect on MMP-2 activity reduction in MDA-MB-231. In conflict with our results, other groups have observed that silencing Foxp3 expression in MCF7 luminal BRCA cells, which have high Foxp3 expression, increases their migratory capacity [28,69]. Conversely, it was reported that forced overexpression of Foxp3 in cells with low expression of Foxp3 decreased their migratory capacity [28]. Our results indicate that blockade of Foxp3 failed to affect the migration of MCF7 cells but exerted a strong inhibitory effect on the migration of HER2+ LM3 and TNBC MDA-MB-231 cells, suggesting that Foxp3 may elicit differential tumor intrinsic effects depending on the tumor molecular subtype. In addition, it is possible that retaining Foxp3 in the cytoplasm (by the inhibition of its nuclear translocation with P60) reveals the effects of the sustained interaction of Foxp3 with cytoplasmic signaling pathways.; this is unlike what happens when the expression of both nuclear and cytoplasmic Foxp3 is erased from the cell via shRNA. In fact, in hepatocellular carcinoma, mRNA alterations in the FKH region of Foxp3 influenced its subcellular localization and altered its function [70]. To accomplish its functions, Foxp3 forms a multimeric complex with a myriad of other molecules, which includes 350 proteins [71]. Thus, retaining Foxp3 in the cytoplasm, rather than impairing its nuclear functions, could result in antitumor effects. The N-terminal region of the protein was associated with the tumor-suppressor activity of Foxp3 [72,73]. In this way, P60 interaction with the intermediate leucine zipper domain inhibits Foxp3 homodimer formation and immune-suppressor activity, but it does not block other interactions [13]. Therefore, our results suggest that Foxp3 blockade with P60 not only inhibits Tregs function but also promotes antitumor activity within BRCA cells. 

In order to improve the local availability of P60 in BCRA we developed an Adv encoding this peptide. Although E1/E3-deleted adenoviral vectors have been evaluated in cancer patients in several clinical trials over the years, showing excellent toxicity profiles, there are limitations that could affect the efficacy of these vectors. The high immunogenicity of these vectors leads to transient transgene expression due to a robust anti-adenoviral immune response [74]. High-capacity vectors are devoid of all adenoviral genes and thus the immune system cannot detect them, allowing for long-term expression even in animals preimmunized against adenoviruses [75]. In addition, they have proved to exert very robust transgene expression in vivo and in vitro in normal and neoplastic tissues [76]. The absence of adenoviral genes greatly increases the cloning capacity of these vectors, allowing the introduction of several therapeutic transgenes within the same vector or the inclusion of regulatory sequences that control transgene expression, i.e., inducible promoters and their inducers and repressors. These vectors are excellent candidates to deliver therapeutic transgenes in cancer and other chronic diseases once they have been proven to be safe in clinical settings. Advs have been used in BCRA, leading to antitumor immunity by the immunization with a nonfunctional HER2 molecule, as well as the local overexpression of the proinflammatory molecule IL-12 or proapoptotic molecules [77]. Likewise, the intranasal administration of Advs allows high transduction efficiency in the lung, thus it has been proposed to treat lung metastases [78]. Recently, an Adv encoding for a hypoxia-inducible factor (HIF)-3α4 and CD44 decoy protein system that is activated by Notch ligands has demonstrated efficacy in murine models of human TNBC subtypes [79]. Here, we observed robust transduction efficiency of Ad.dTomato and Ad.P60 in murine BRCA cells both in vitro and in vivo. Although both vectors readily transduced breast cancer cells, the percentage of positive cells was lower when they were transduced with Ad.P60. This could be due to the fact that dTomato is expressed directly under the control of the CMV in the control vector, whereas in the Ad.P60 vector the dTomato sequence follows an internal ribosome entry site that connects it to the P60 sequence. Although this P60-IRES-dTomato sequence expression is also controlled by the CMV promoter, the expression of the subsequent (dTomato) is usually lower that the first one (P60) [80]. The lower transduction efficiency observed for Ad.P60 could also be related to the cytotoxic effect of P60 in these cells. Our findings indicate that both the P60 peptide and Ad.P60 exert direct antitumor effects, reducing proliferation and increasing apoptosis in these cells. In fact, the images of transduced cells show that there are fewer cells per field when cells were transduced with Ad.P60 than Ad.dTomato. Ad.P60 exerted direct antitumor effects in vitro and in vivo that were similar or even greater than those observed when using the P60 peptide [22]. The stable expression of the peptide may cause the increased local bioavailability of the peptide in the TME. Our results using conditioning medium from Ad.P60-transduced tumor cells, indicate that Ad.P60 elicits bystander effect by stimulating leukocyte proliferation. This could be due to the release of P60 into the culture medium or the result of changes in the profile of pro- and anti-inflammatory molecules released by transduced tumor cells. In fact, P60 [22] and pl.P60 inhibited the release of IL-10 in BRCA cells, suggesting that this treatment could change the composition of the TME. In line with these observations, Ad.P60 decreased tumor Treg infiltration. These results agree with our previous data using systemic administration of the P60 peptide [22]. It is important to mention that, unlike other strategies, P60 allows the blockade of Foxp3 without depleting Tregs [13]. In fact, we observed no changes in the content of Tregs in the spleen. This is an advantageous feature as Treg depletion has been shown to be counteracted by the conversion of CD4^+^ T cells to CD4^+^CD25^+^Foxp3^+^ in a homeostatic system, a mechanism that restores immune tolerance [81]. Taken together, our results suggest that local administration of Ad.P60 could promote a more immunologically active TME. These mechanisms, in addition to the inhibition of the tumor intrinsic effect of Foxp3, could be mediating the antitumor efficacy of Ad.P60, which decreased tumor growth in vivo in BRCA preclinical models and reduced spontaneous lung metastases. This strategy is much simpler than administering the P60 peptide systemically and features the characteristic versatility of Advs, which allow the incorporation of tumor-specific or inducible promoters to restrict the expression of the therapeutic transgene spatially and temporally. 

In conclusion, our results indicate that inhibition of Foxp3, by inhibiting Treg function and promoting antitumor activity in BRCA cells, could facilitate not only the efficacy of immunotherapeutic strategies but could also improve the response to chemotherapy in BRCA. Our findings warrant further evaluation of these combined treatments in relevant models of breast cancer. The possibility of delivering the P60 peptide using Advs could facilitate these treatments. The combination of multiple strategies could help patients with resistant tumors that lack therapeutic alternatives.

## Data Availability

Not applicable.

## Supplementary Materials

The following supporting information can be downloaded at: https://www.mdpi.com/article/10.3390/v15091813/s1, Supplementary Materials and Methods: Flow cytometry, Immunocytochemistry, Viral vector characterization; Supplementary Figures; Figure S1: Chemotherapeutic drug concentration-response curves in BRCA; Figure S2: Full-length gel shows MMP-9 and MMP-2 activity in EO771 BRCA cells; Figure S3: Full-length gel shows MMP-9 and MMP-2 activity in LM3 BRCA cells; Figure S4: Foxp3 tumor intrinsic effects: endothelial cell migration; Figure S5: Foxp3 tumor intrinsic effects in MCF-7 human luminal BRCA cells: migration; Figure S6 Full-length gel shows MMP-9 and MMP-2 activity in MDA-MB-231 BRCA cells; Figure S7: Content of CD4+ and CD8+ lymphocytes in tumor and spleen after intratumoral administration of Ad.P60.
