# Supplementary material for: Targeting FOXP3 Tumor-Intrinsic Effects Using Adenoviral Vectors in Experimental Breast Cancer
Source: Viruses. 2023 Aug 25;15(9):1813. doi: 10.3390/v15091813 (PMC10537292; doi:10.3390/v15091813)
Supplement: Supplementary file 1 [file viruses-15-01813-s001.zip › viruses-2549026-supplementary.pdf]

SUPPLEMENTARY DATA

SUPPLEMENTARY MATERIALS AND METHODS

Flow cytometry

Mononuclear cells were purified from the spleen and tumors as previously described [1,2]. Briefly, spleens were removed and smashed to release splenocytes. Red blood cells were lysed by incubating the preparation in 3 volumes of ice-cold ammonium-chloride-potassium solution (ACK, 0.15 M NH_4Cl, 10 mM $KHC0_3$, 0.1 mM Na_2EDTA) for 3 min and then washed with supplemented RPMI. In order to purify tumor infiltrating immune cells, tumors were dissected and disaggregated with a glass homogenizer. Mononuclear cells were obtained from the interphase of 30-90% Percoll gradient as previously described [2].

Mononuclear cells were washed with flow cytometry buffer (1% FBS) and incubated with anti-CD45, anti-CD8 and anti-CD4 for 1 h (Biolegend, San Diego, CA, Cat #103105, 100705, 100539, respectively, San Diego, CA). After washing, cells were fixed with True-Nuclear™ Transcription Factor Buffer Set according to manufacturer instructions (BioLegend, San Diego, CA, Cat #424401). Then, cells were stained with anti-Foxp3 antibody (eBioscience, MA, Cat# 17-5773-82), washed and maintained in 0.1% sodium azide in PBS at 4°C until analyzed by flow cytometry.

Tumor cells growing in monolayer were harvested with trypsin-EDTA. After washing, cells were fixed with True-Nuclear™ Transcription Factor Buffer Set according to manufacturer instructions (BioLegend, San Diego, CA, Cat #424401). Then, cells were stained with anti-Foxp3 antibody (eBioscience, MA, Cat# 17-5773-82), washed and maintained in 0.1% sodium azide in PBS at 4°C until analyzed by flow cytometry. Data obtained from flow cytometry analysis were processed using FlowJo v10 software.

Immunocytochemistry

Cells were fixed and permeabilized using transcription factor buffer set (BioLegend; cat # 424401). To block non-specific binding sites, 10% goat serum was used in permeabilizing buffer for 1 hour. Subsequently, cells were incubated with an anti-Foxp3 antibody (1:50, BioLegend Cat# 320102) in permeabilizing buffer overnight. The next day, the cells were washed and incubated with an anti-mouse IgG antibody (1:100, Thermo Fisher Scientific Cat# A11029). Finally, cells were stained with DAPI (4',6-diamidino-2-phenylindole) used at a concentration of 5 μg/ml and mounted on slides with Vectashield (Vector Laboratories, Inc., Newark, CA). Visualization of cells was performed using a fluorescent light microscope (Axiophot; Carl Zeiss, Jena, Germany). Negative controls were made by incubating cells without the primary antibody.

PFA-fixed EO771 tumors were dissected and 50 μm cryostat sections were made. Immunohistochemistry was performed using free-floating technique in which tumors sections were incubated in liquid throughout the process. Sections were incubated with DAPI for 10 min and mounted in anti-fade mounting medium Vectashield.

Viral vector characterization

Murine breast cancer cells LM3 and EO771, or human MDA-MB-231 were incubated *in vitro* with Ad.dTomato or Ad.P60 at MOI 200 for 48 h. Transduction efficiency was evaluated by detecting dTomato-positive cells by fluorescence microscopy. The effect on the viability of

EO771 cells, and the *bystander effect* of CM on the proliferative capacity of splenocytes co-activated with 5 μg/ml of anti-CD3 (clone 145-2C11) (BD, Cat# 553057) and with 1 μg/ml anti-CD28 (clone 37.51) (BD, Cat# 553294) antibodies were evaluated.

SUPPLEMENTARY FIGURES AND LEGENDS

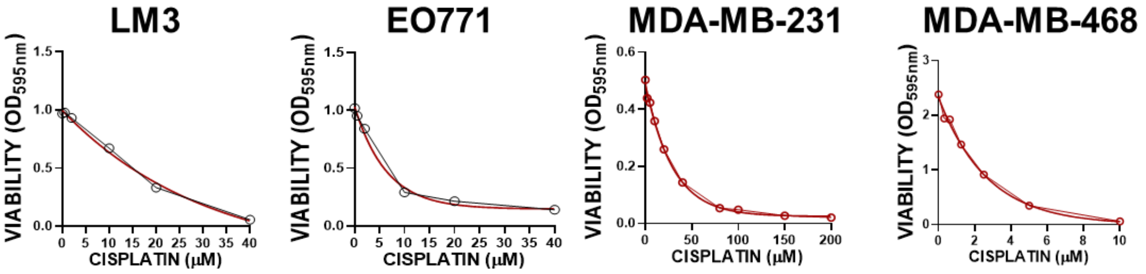

Supplementary Figure S1. Chemotherapeutic drug concentration-response curves in BRCA cells. BRCA cells were incubated with different concentrations of cisplatin for 72 h. Cell viability was assessed by MTT assay. Dotted lines connect the mean ± SEM at each concentration. Full lines: multiple regression analysis

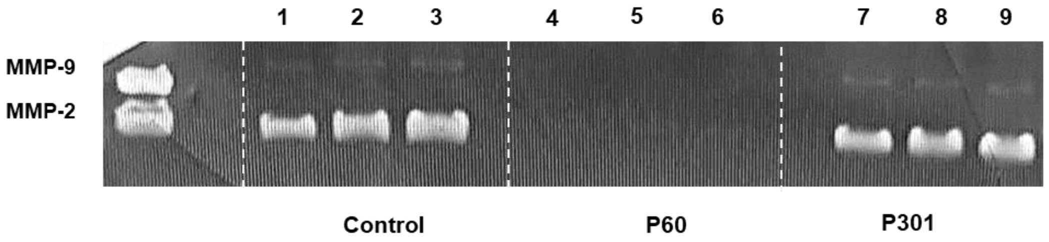

Supplementary Figure S2: Full-length gel shows MMP-9 and MMP-2 activity in EO771 BRCA cells. SDS-PAGE gelatine zymography of conditioned media from mice EO771 BRCA cells control (1-3) or incubated with 50 μM P60 (4-6) or P301 (7-9) for 48 h. Gels were stained with Coomassie blue and bands were analysed by densitometry with ImageJ software. Zymographic activity was expressed as percentage in relation to a standard internal sample (MMP-9 and MMP-2) that saturates at a density of 50%.

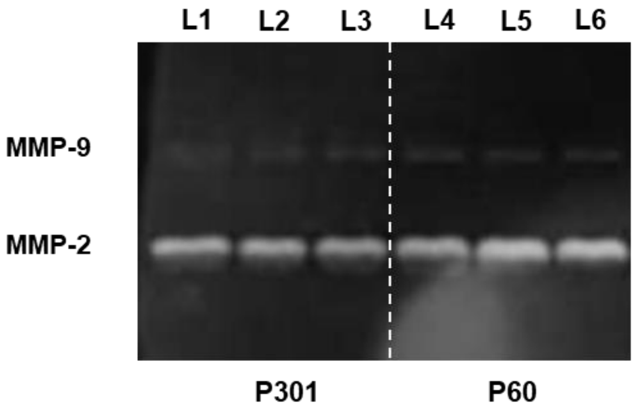

Supplementary Figure S3: Full-length gel shows MMP-9 and MMP-2 activity in LM3 BRCA cells. SDS-PAGE gelatine zymography of conditioned media from mice LM3 BRCA cells incubated in the presence of 50 μM P301 (L1-L3) or P60 (L4-L6) for 48 h. Gels were stained with Coomassie blue and bands were analysed by densitometry with ImageJ software. Zymographic activity was expressed as percentage in relation to a standard internal sample (MMP-9 and MMP-2) that saturates at a density of 50%.

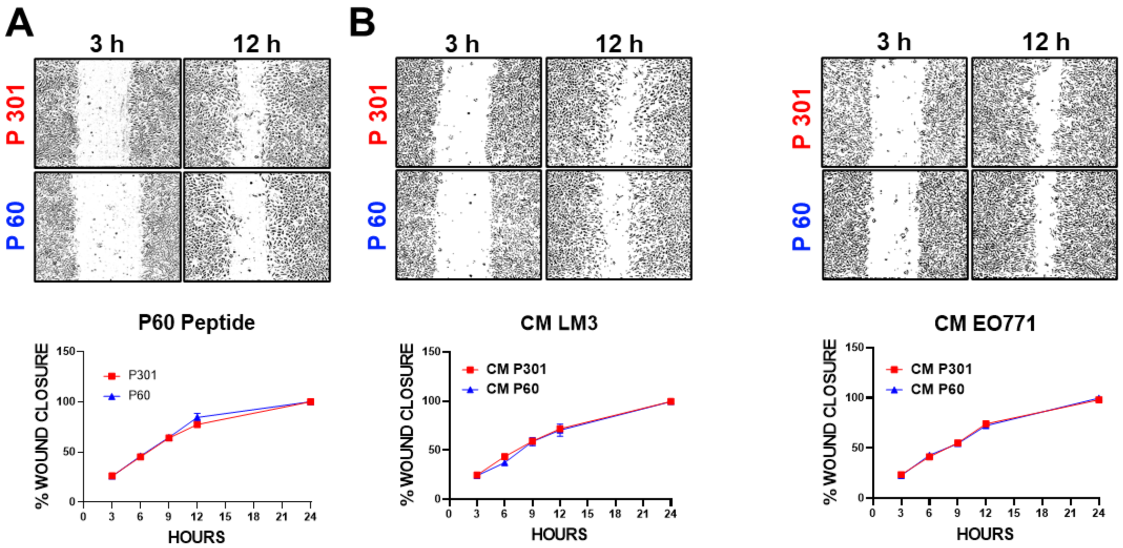

Supplementary Figure S4. Foxp3 tumor intrinsic effects: endothelial cell migration. (A) HER2+ LM3 cells or (B) TNBC EO711 cells were incubated with peptide P301 or P60 (50 μM) for 48 h and the conditioned medium (CM) was collected. The migratory capacity evaluated by the wound assay was assessed in endothelial cells EA.hy.926 incubated with P60 or P301

peptides or with CM of LM3 or EO771 cells for 24 h. Representative microphotographs at 3 and 12 h of incubation are shown.

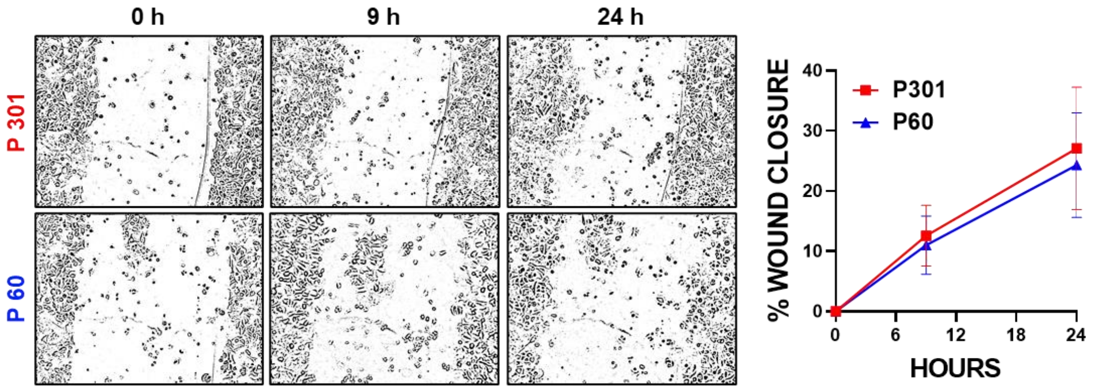

Supplementary Figure S5. Foxp3 tumor intrinsic effects in MCF-7 human luminal BRCA cells: migration. MCF-7 cells were incubated with P60 or P301 (50 μM) for 24 h. Migration was assessed by the wound assay. Representative microphotographs at 3 and 12 h of incubation are shown.

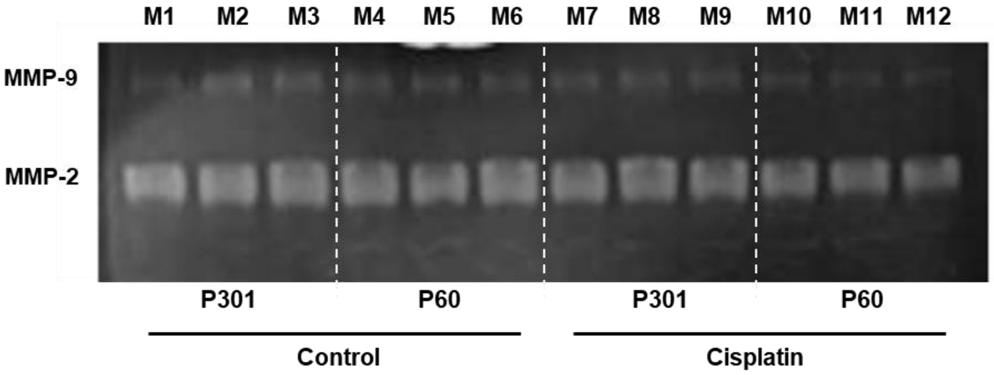

Supplementary Figure S6: Full-length gel shows MMP-9 and MMP-2 activity in MDA-MB-231 BRCA cells. SDS-PAGE gelatine zymography of conditioned media from human MDA-MB-231 BRCA cells incubated with 50 μM of P301 (M1-M3, M7-M9) or P60 (M4-M6, M10-M12) for 48 h in presence or absence of cisplatin (40 μM). Gels were stained with Coomassie blue, and bands were analysed by densitometry with ImageJ software. Zymographic activity was expressed as percentage in relation to a standard internal sample (MMP-9 and MMP-2) that saturates at a density of 50%.

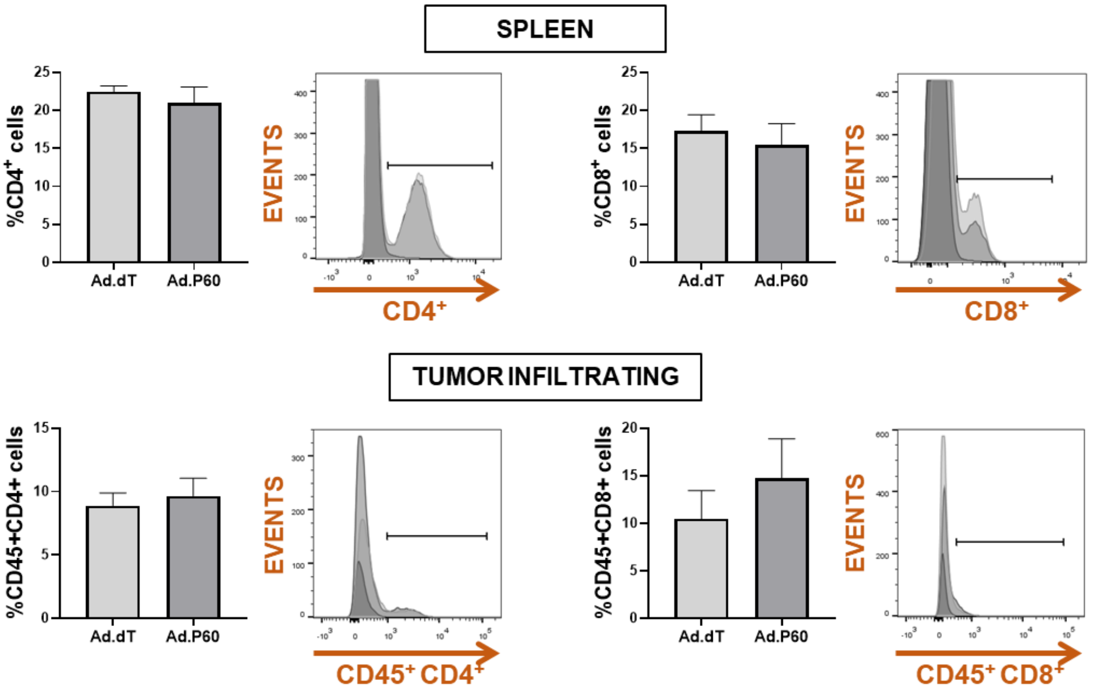

Supplementary Figure S7. Content of CD4+ and CD8+ lymphocytes in tumor and spleen after intratumoral administration of Ad.P60. Foxp3-GFP transgenic C57Bl/6 mice were administered subcutaneously with 200,000 syngeneic EO771 cells. When tumor volume reached 500 mm^3, mice were treated with an i.t. injection of 6.3x10^{7}UFP of Ad.dT or Ad.P60. Spleen and tumor-infiltrating CD4+ and CD8+ T cells were quantified by flow cytometry 8 d after inoculation.

1. Jacob, J.B.; Kong, Y.C.; Meroueh, C.; Snower, D.P.; David, C.S.; Ho, Y.S.; Wei, W.Z. Control of Her-2 tumor immunity and thyroid autoimmunity by MHC and regulatory T cells. *Cancer Res* **2007**, *67*, 7020-7027, doi:10.1158/0008-5472.Can-06-4755.
2. Liyanage, U.K.; Moore, T.T.; Joo, H.G.; Tanaka, Y.; Herrmann, V.; Doherty, G.; Drebin, J.A.; Strasberg, S.M.; Eberlein, T.J.; Goedegebuure, P.S.; et al. Prevalence of regulatory T cells is increased in peripheral blood and tumor microenvironment of patients with pancreas or breast adenocarcinoma. *J Immunol* **2002**, *169*, 2756-2761, doi:10.4049/jimmunol.169.5.2756.
